# COVID‐19 in pregnancy: Risk of adverse neonatal outcomes

**DOI:** 10.1002/jmv.25959

**Published:** 2020-06-19

**Authors:** Aman Mehan, Ashwin Venkatesh, Milind Girish

**Affiliations:** ^1^ School of Clinical Medicine University of Cambridge Cambridge UK

**Keywords:** coronavirus < virus classification, pandemics < epidemiology, respiratory tract < pathogenesis, vertical transmission < epidemiology

To the Editor,

The outbreak of coronavirus disease 2019 (COVID‐19) is a major public health concern and increasing cases of infection are being reported during pregnancy. In the *Journal of Medical Virology*, Chen et al report clinical features and outcomes of pregnant patients with COVID‐19 in the Maternal and Child Hospital of Hubei Province, China.^1^ The authors present five patients (aged 25‐31) with COVID‐19 at term gestation (38‐41 weeks), in whom delivery was uneventful and led to favorable perinatal outcomes. We write to highlight that the small cohort in Chen et al is not representative of the overall literature to date. Moreover, whilst infection at term might be relatively inconsequential, a growing body of evidence now points towards an association between preterm maternal severe acute respiratory syndrome‐coronavirus 2 (SARS‐CoV‐2) infection, preterm delivery and adverse neonatal outcomes, which has been under‐addressed.

Although none of the patients in the study by Chen et al^1^ delivered preterm, our review of the literature highlights several instances in which preterm delivery together with the outcomes for both mother and neonate have been documented (Table 1). For instance, Liu et al report that 6 out of 13 (46%) hospitalized pregnant patients with COVID‐19 delivered preterm (between 32 and 36 weeks' gestation).^4^ One patient developed pneumonia and septic shock, requiring intensive care admission and emergency caesarean section, unfortunately yielding a stillbirth. In another study of 10 neonates born to mothers with COVID‐19 pneumonia, six were preterm and of low birth weight.^3^ Whilst none tested positive for SARS‐CoV‐2 at 72 hours post‐birth, all preterm neonates had a pediatric critical illness score below 90, one of whom died with four remaining hospitalized. No underlying maternal disease or neonatal infection was reported, thus preterm maternal COVID‐19 may independently predict poor neonatal outcome. A case‐control study recently published by Li et al^9^ further strengthens the evidence base for this association, highlighting a significantly increased incidence of preterm delivery in cases of COVID‐19 than controls, which the authors attributed to gestational complications, such as premature rupture of membranes and placental bleeding.

**Table 1:** Studies reporting preterm deliveries (as of 10/4/2020)

Parameters	Findings							
Study	Chen et al^2^	Zhu et al^3^	Liu et al^4^	Zeng et al^5^	Zhang et al^6^	Chen et al^7^	Liu et al^8^	Li et al^9^
Study period	20.1.20‐31.1.20	20.1.20‐5.2.20	8.12.19‐5.2.20	Jan‐Feb	30.01.20‐17.02.20	30.01.20‐23.2.20	20.1.20‐ 10.2.20	24.1.20‐ 29.2.20
Confirmed SARS‐CoV‐2+ pregnant women	9	9	13	33	16	17	15	16
Time point of symptom presentation								
1st trimester	0	0	0	NR	0	NR	1	0
2nd trimester	0	0	2	NR	0	NR	2	0
3rd trimester (before birth)	9	6	11	NR	16	NR	12	4
After delivery	0	3	0	NR	0	NR	0	8
Maternal morbidity with COVID‐19								
Asymptomatic	0	0	1	NR	NR	9	2	4
Symptomatic	9	9	11	NR	NR	8	13	12
Severe	0	0	1	NR	1	0	0	0
Total number that delivered	9	9	13	33	16	17	11	16
Cesarean delivery	9	7	5	26	16	17	10	14
Pregnancy outcome								
Miscarriage	0	0	1	0	0	0	0	0
Preterm delivery	4	5	6	4	1	3	3	4
Term delivery	5	4	6	29	15	14	8	12
Recovered without delivering	0	0	0	0	0	0	NR	0
Maternal mortality	0	0	0	0	0	0	0	0
Number of neonates included in study	9	10	13	33	10	17	11	17
Neonatal adverse outcome								
Pneumonia	0	4	NR	3	3 (bacterial)	0	NR	NR
Low birthweight	2	6	NR	NR	NR	0	NR	3
SGA	NR	2	NR	3	NR	NR	NR	NR
NRDS	0	2	NR	4	0	0	NR	0
Preterm neonatal adverse outcome								
Pneumonia	0	2/6	NR	1/4	NR	0	NR	NR
Low birthweight	2/4	6/6	NR	NR	NR	0/3	NR	NR
SGA	NR	0	NR	NR	NR	NR	NR	NR
NRDS	0	0	NR	NR	NR	0	NR	NR
Neonatal death	0	1/6	1/6	0	0	0	0	0
Confirmed SARS‐CoV‐2+ neonates	0	0	0	3	0	0	NR	0
Vertical transmission reported	0	^a^	0	^a^	0	0	NR	0
Horizontal transmission reported	0	0	0	3	0	0	NR	0
Symptomatic	0	0	0	3	0	0	NR	0
Newborn mortality	0	1/10	1/13	0	0	0	0	0

Abbreviations: COVID, coronavirus disease; NR, not reported; NRDS, neonatal respiratory distress syndrome; SARS‐CoV‐2, severe acute respiratory syndrome‐coronavirus 2; SGA, small for gestational age.

^a^ Vertical transmission not ruled out.

In Chen's et al^1^ cohort, all SARS‐CoV‐2+ women were asymptomatic before delivery, with two developing mild symptoms postpartum, and the majority delivered vaginally. The greater adoption of C‐section in the studies we examined was influenced by: perceived risk of vertical transmission, the lack of available negative pressure operating rooms, deteriorating maternal symptoms, or indeed conventional obstetric indications. We also noted that severe COVID‐19 associated maternal morbidity was documented in three studies, which yielded adverse neonatal outcomes as one might expect. However, interestingly the majority of women generally exhibited minor symptoms or were asymptomatic ‐ consistent with Chen et al^1^ ‐ yet even in such cases preterm birth was reported. Thus, maternal SARS‐CoV‐2 infection may be an independent risk factor for preterm birth, regardless of symptom severity, though the mechanism remains to be clarified. Moreover, asymptomatic infections may present a considerable challenge for managing transmission risk in obstetric units. Thus, studies evaluating whether universal testing offers cost‐effective improvements in clinical outcomes would be beneficial.

Whilst the neonates in Chen's et al^1^ cohort were healthy and of normal birthweight, the literature highlights that in preterm deliveries, adverse neonatal outcomes can be expected. In the studies examined, this most commonly manifested as pneumonia and low birth weight and was concurrent with the death of two neonates. Follow up studies would be useful to assess whether longer‐term outcomes of neonates born preterm to SARS‐CoV‐2+ mothers differ from preterm births without an associated maternal SARS‐CoV‐2 infection and may also illustrate any eventual consequences of maternal COVID infection that were absent at birth.

Despite acknowledging its possibility, Chen et al^1^ did not test for vertical transmission and we would like to emphasize that vertical transmission should not be excluded as a potential mechanism. Indeed, vertical transmission has been under‐tested and sparsely reported. This may relate to the challenges of testing neonates, since only a minority are symptomatic, and both oral and nasal swab testing have low sensitivity.^3^, ^9^, ^10^ However, emerging evidence of a neonate with elevated IgM antibodies to SARS‐CoV‐2 highlights the possibility of this phenomenon.^10^ It is plausible that other existing reports of neonatal early onset infection may also represent vertical transmission. Notably, Zeng et al report of a neonate infected with SARS‐CoV‐2 that developed severe clinical sequelae.^5^ Although the authors attributed this outcome to prematurity, asphyxia and sepsis, the compounding pathological contributions of the virus to prematurity, and of potential vertical transmission to adverse neonatal outcomes, require further elucidation. Recent research has revealed a high expression of the SARS‐CoV‐2 receptor, ACE2, and the serine protease for virus spike protein priming, TMPRSS2, in maternal‐fetal interface cells. This may represent a potential mechanism for vertical transmission that warrants further investigation.^11^

Overall, we thank Chen et al^1^ for highlighting issues surrounding maternal SARS‐CoV‐2 infection at term but wish to draw attention to the prevalence of preterm maternal SARS‐CoV‐2 infection and its association with premature neonatal delivery and adverse outcomes. This warrants that obstetric and neonatal units institute anticipatory precautions when presented with a SARS‐CoV‐2 positive pregnancy before term.

## CONFLICT OF INTERESTS

The authors declare that there are no conflicts of interest.

## References

[1] Chen S , Liao E , Shao Y . Clinical analysis of pregnant women with 2019 novel coronavirus pneumonia. J Med Virol. 10.1002/jmv.25789 PMC7228212 32222119

[2] Chen H , Guo J , Wang C , et al. Clinical characteristics and intrauterine vertical transmission potential of COVID‐19 infection in nine pregnant women: a retrospective review of medical records. Lancet. 2020;395(10226):809‐815. 10.1016/S0140-6736(20)30360-3 32151335 PMC7159281

[3] Zhu H , Wang L , Fang C , et al. Clinical analysis of 10 neonates born to mothers with 2019‐nCoV pneumonia. Transl Pediatr. 2020;9(1):51‐60. 10.21037/tp.2020.02.06 32154135 PMC7036645

[4] Liu Y , Chen H , Tang K , Guo Y . Clinical manifestations and outcome of SARS‐CoV‐2 infection during pregnancy. J Infect. 2020 10.1016/j.jinf.2020.02.028 PMC7133645 32145216

[5] Zeng L , Xia S , Yuan W , et al. Neonatal early‐onset infection with SARS‐CoV‐2 in 33 neonates born to mothers with COVID‐19 in Wuhan, China. JAMA Pediatr. 2020 10.1001/jamapediatrics.2020.0878 PMC7099530 32215598

[6] Zhang L , Jiang Y , Wei M , et al. Analysis of the pregnancy outcomes in pregnant women with COVID‐19 in Hubei Province. Zhonghua Fu Chan Ke Za Zhi. 2020;55(0):E009 10.3760/cma.j.cn112141-20200218-00111 32145714

[7] Chen R , Zhang Y , Huang L , Cheng B‐H , Xia Z‐Y , Meng Q‐T . Safety and efficacy of different anesthetic regimens for parturients with COVID‐19 undergoing Cesarean delivery: a case series of 17 patients. Can J Anaesth. 2020 10.1007/s12630-020-01630-7 PMC7090434 32180175

[8] Liu D , Li L , Wu X , et al. Pregnancy and perinatal outcomes of women with coronavirus disease (COVID‐19) pneumonia: a preliminary analysis. AJR Am J Roentgenol. 2020:1‐6. 10.2214/AJR.20.23072 32186894

[9] Li N , Han L , Peng M , et al. Maternal and neonatal outcomes of pregnant women with COVID‐19 pneumonia: a case‐control study. Clin Infect Dis. 10.1093/cid/ciaa352 PMC7184430 32249918

[10] Dong L , Tian J , He S , et al. Possible vertical transmission of SARS‐CoV‐2 from an infected mother to her newborn. JAMA. 2020 10.1001/jama.2020.4621 PMC7099527 32215581

[11] Li M , Chen L , Zhang J , Xiong C , Li X . The SARS‐CoV‐2 receptor ACE2 expression of maternal‐fetal interface and fetal organs by single‐cell transcriptome study. PLoS One. 2020;15:15. 10.1371/journal.pone.0230295 PMC7161957 32298273

